# Elites' perceptions of women's representation in the Omani media

**DOI:** 10.3389/fsoc.2025.1724294

**Published:** 2025-11-27

**Authors:** Sumaiya Al-Wahaibi

**Affiliations:** 1 Department of Cross-Cultural and Regional Studies, Faculty of Humanities, Copenhagen University, Copenhagen, Denmark; 2 Faculty of Business, Sohar University, Sohar, Oman

**Keywords:** gender, media analysis, GCC (Bahrain; Saudi Arabia; Kuwait; United Arab Emirates; Qatar; Oman; Arabian Gulf; Persian Gulf), media portrayal of women, neopatriarchy, symbolic annihilation, state feminism

## Abstract

**Introduction:**

This study investigates how Omani elites perceive women's representation in national media and how structural, cultural, and political forces shape gender narratives in the Sultanate. It explores how women's visibility is instrumentalized to project modernization while maintaining patriarchal authority, situating the Omani case within broader Gulf Cooperation Council (GCC) media-gender dynamics.

**Methods:**

The research draws on 38 semi-structured elite interviews conducted between 2021 and 2025 with parliamentarians, journalists, academics, activists, and government officials. Interviews were analyzed using reflexive thematic analysis within the frameworks of state feminism, neopatriarchy, and symbolic annihilation, complemented by media observation and document analysis to triangulate findings.

**Results:**

Findings reveal that women's visibility in Omani media is predominantly symbolic and event-based, emphasizing ceremonial achievements and depoliticized empowerment. Elites describe a system where modernization coexists with patriarchal control, producing what can be termed “modernization without liberation.” Media governance and restrictive laws-such as those protecting the “majesty of the state,” limit editorial independence and suppress rights-based gender discourse. Women are often silenced or stereotyped, with their lived realities of discrimination and inequality omitted from mainstream narratives.

**Discussion and conclusions:**

The study argues that women's representation in Omani media functions as a technology of legitimacy within an authoritarian developmental model that merges inclusionary rhetoric with moral governance. Moving beyond symbolic visibility requires reforms that strengthen media autonomy, institutionalize women's participation in decision-making, and expand digital pluralism. Such measures could transform Omani media from a vehicle of state image management into a platform for genuine dialogue, justice, and equality, contributing to wider debates on gender, media, and state-society relations in the GCC.

## 1 Introduction

Media plays a fundamental role in shaping societal narratives and public perceptions, particularly concerning gender relations, roles, and women's issues. In Oman, the portrayal of women in media reflects a delicate balance between tradition and modernization, with elite perceptions significantly influencing both the scope and framing of these narratives. This paper asks: how do Omani elites perceive women's representation in the media, and what does this reveal about the structural and cultural constraints shaping such portrayals?

This research is original in that it provides the first systematic elite-based study of media–gender representation in Oman, drawing on in-depth interviews with policymakers, journalists, academics, and civil society figures. The study contributes to broader debates on GCC state–society relations, where the media operates as both a vehicle of state-led modernization and a site of negotiation for contested gender norms (Ehteshami and Wright, 2007, p. 911; Mellor et al., 2011, pp. 22–23). While the Omani media landscape is characterized by strong state oversight, it also contains spaces of negotiation and subtle contestation. Recent years have seen the rise of digital activism, independent online commentary, and women-led community initiatives that engage issues of gender and social justice beyond formal media channels. Although these movements remain limited in scale and visibility, they underscore that Omani society is not monolithically state-dominated but rather shaped by ongoing, if restrained, dialogues between authority and agency.

The theoretical framework guiding this analysis draws on three interlinked perspectives. First, state feminism highlights how Gulf governments selectively promote women's achievements to project an image of progress, while avoiding deeper structural reforms that would challenge existing hierarchies (Charrad and Zarrugh, 2015, p. 4; Al-Rasheed, 2013, p. 9). Second, neopatriarchy captures the coexistence of modern state institutions with entrenched patriarchal norms, where women's visibility in public life is celebrated symbolically but curtailed substantively (Sharabi, 1988, pp. 7–9). Finally, symbolic annihilation explains how women are marginalized in media either through absence, stereotyping, or trivialization, reinforcing gender inequalities (Tuchman, 1978, p. 8; Gallagher, 2014, p. 118). Together, these perspectives help explain why women's empowerment is often portrayed as symbolic and service-oriented rather than rights-based or transformative.

By focusing on elite perceptions, the study illuminates how cultural and religious norms, such as the emphasis on women as caregivers, moral custodians, or symbols of national progress, shape media representations that sidestep contentious issues such as inheritance, divorce, or citizenship rights (Azri, 2013, p. 10; Al-Lamki, 2007, pp. 61–62). In doing so, the research contributes to understanding the intersection of tradition, modernity, and gender reform in the Gulf, and provides a window into the political economy of media governance in Oman. The paper proceeds as follows: Section 2 reviews the literature on media, gender, and governance in the Gulf; Section 3 outlines the methodology; Section 4 presents the findings based on elite interviews; Section 5 discusses these findings in light of the theoretical framework; and Section 6 concludes with implications for policy and media practice.

## 2 Literature review

### 2.1 Media and the state in Oman and the GCC

Across the Gulf, media has historically operated as a vehicle for state-led modernization, projecting women's empowerment as part of national development agendas within carefully managed political and cultural limits. In Saudi Arabia, media platforms have celebrated reforms symbolically while avoiding structural critique (Al-Rasheed, 2013, p. 9). Similarly, in Kuwait and the UAE, tribal and heritage narratives, including gendered visibility, have been strategically mobilized to reinforce state legitimacy and modernization credentials (Al-Sharekh and Freer, 2022, pp. 9–20). Ghobain et al. (2024, pp. 3, 10–13) found that Saudi newspaper portrayals of women's military enlistment framed reform as patriotic progress yet avoided discussing entrenched inequalities, transforming empowerment into a performative act of loyalty. In the UAE, Al Obeidli's ethnographic study of newsrooms shows that women journalists navigate a dual matrix of overt state regulation and subtler gendered expectations governing professional behavior and tone (Buscemi et al., 2024, p. 12). Across the GCC, these examples reveal a persistent gap between rhetorical inclusion and substantive empowerment.

Oman represents a more cautious trajectory; one deeply shaped by its post-1970 state-building experience. The Dhofar War (1965–1975) was pivotal in defining the modern Omani state and its media infrastructure. During the conflict, Sultan Qaboos's government used newly nationalized newspapers such as Oman Daily and Al-Watan and radio broadcasts to promote narratives of unity, stability, and modernization in opposition to the Dhofari insurgency (Valeri, 2017, pp. 144–146). Communication became a tool of consolidation, linking citizens in far-flung regions to a shared national identity and projecting the Sultan as a reformist leader committed to progress and development. This period institutionalized the notion that media served not merely to inform but to build the nation. It also established the precedent for what would later evolve into state feminism: the state's selective promotion of women's education, employment, and public participation as evidence of modernization and national cohesion. Following the war, successive legislation, including the 1975 Publications Law, the 1984 Press and Publications Law, and the 1996 Basic Statute, codified government control over the press and reinforced its role as a developmental instrument (Kechichian, 1995, p. 222; Bahgat, 2023, p. 97). Even with the introduction of private outlets in the 2000s, editorial independence remained limited; the 2016 closure of Azamn newspaper after reports on judicial corruption exemplified the boundaries of permissible critique (Freedom House, 2017). Furthermore, family-focused television programs that emerged after the 1990s, *Shu'*ū*n ‘*$\bar{\text{A}}$*iliyya* (1999–2008), *Shaq*ā'*iq* (2006–2007), *Hay*ā*t'kum* (2013–2016), and *Kay*ā*n* (2021–2024), continued this legacy. They showcased women's participation yet operated largely as soft-cultural platforms rather than forums for critical debate, echoing a wider GCC pattern where women are presented as symbols of national progress while issues such as inheritance, guardianship, and workplace discrimination remain absent (Sonbol, 2012, p. 7; Dhaheri, 2009, p. 282). As Bronstein (2005, p. 173) observes, such selective portrayals exemplify a constructionist paradigm in which media both reflects and produces normative gender hierarchies.

Before 2020, studies addressing women and media in Oman were scarce but provided essential groundwork for understanding gendered representation under state-led modernization. Subhi and Smith (2019, pp. 93, 104) found that tribalism and religiosity constrained support for women candidates in the Majlis al-Shura elections, reinforcing patriarchal attitudes toward leadership. Linguistic and digital-communication research identified subtle gendered norms in expression and interaction, reflecting broader social hierarchies (Al-Rashdi, 2018, p. 118). Regional advertising studies revealed the persistence of domestic and decorative portrayals of women (Khalil and Dhanesh, 2020, p. 674), while experimental work on emoji interpretation demonstrated enduring gender bias in communication (Butterworth et al., 2019, pp. 2–4). Together, these works show that symbolic inclusion rarely translated into substantive participation.

After 2020, scholarship diversified to examine policy, production, and audience dynamics. Al-Talei (2021, 2023) argues that official norms of neutrality and respectability depoliticize women's coverage in print and broadcast media. Policy analyses and practitioner interviews reveal how risk-aversion and regulatory oversight limit portrayals of female candidates (Al-Baloushi, 2022, p. 1). Studies on digital participation link heavy social-media use to body-image pressures among Omani women (Al Riyami et al., 2024) and show how online platforms simultaneously enable self-expression and reinforce surveillance (Al-Ghabshiya, 2020). Discourse analysis of 2013–2021 news texts suggests that empowerment language remains performative, emphasizing symbolic importance while neglecting structural reform (Al-Dhahli and Al Gheseini, 2024). Production-side studies further demonstrate how newsroom hierarchies, managerial oversight, and technological adaptation constrain critical engagement with gender and social issues. Across these phases, research reveals continuity in the framing of women's empowerment as a matter of respectability and consensus. Omani media's gender discourse has evolved methodologically, from descriptive to analytical and multi-dimensional, but substantively, it continues to privilege state-curated harmony over structural critique. This study builds upon that trajectory by interrogating elite perspectives, the very actors who shape, regulate, and interpret such portrayals, to uncover how symbolic visibility and institutional patriarchy intersect in contemporary Omani media. Tables 1–3 provides a condensed timeline of Omani media reforms and key legislative milestones.

**Table 1:** Media ecosystem over time (1970–2024).^*^

**Year**	**Event/change**	**Minister(s)**	**Media-related laws**
December 1970	Establishment of the first Ministry of Information under the name Ministry of Information, Social Affairs, and Labor	Abdullah bin Mohammed Al-Taei (late author and poet)	–
1972	Separation of Social Affairs and Labor from Information. Information was assigned to the Directorate of Information, later the Directorate of Information and Tourism	–	–
November 17, 1973	Formation of the Ministry of Information and Tourism	His Highness Sayyid Fahd bin Mahmoud Al Said	–
December 8, 1974	Renaming of the Ministry of Information and Tourism to the Ministry of Information and Culture	His Highness Sayyid Fahd bin Mahmoud Al Said	Royal Decree No. (79/74)
June 26, 1975	Issuance of the Publications Law	His Highness Sayyid Fahd bin Mahmoud Al Said	Royal Decree No. (03/75)
November 8, 1976	Issuance of the Law on the Control of Artistic Works	His Highness Sayyid Fahd bin Mahmoud Al Said	Royal Decree No. (45/76)
May 22, 1979	Appointment of Abdulaziz bin Mohammed Al-Rawas as Minister of Information and Youth Affairs		Royal Decree No. (28/79)
May 29, 1980	Establishment of a press and publishing house called “Dar Oman Newspaper,” affiliated to the Ministry of Information and Youth Affairs	Abdulaziz bin Mohammed Al-Rawas	Royal Decree No. (49/80)
May 23, 1982	Renaming of the Ministry of Information and Youth Affairs to the Ministry of Information; Youth Affairs sector moved to the Ministry of Education and Youth Affairs	Abdulaziz bin Mohammed Al-Rawas	Royal Decree No. (40/82)
June 2, 1982	Issuance of the provisions of the Media Sector System in the Ministry	Abdulaziz bin Mohammed Al-Rawas	Royal Decree No. (03/82)
June 6, 1983	Establishment of the Omani Authority for Television Art Production, affiliated to the Ministry of Information	Abdulaziz bin Mohammed Al-Rawas	Royal Decree No. (38/83)
1984	Implementation of the Press and Publications Law, regulating media content and operations	Abdulaziz bin Mohammed Al-Rawas	Press and Publications Law (Royal Decree No. 49/1984)
1996	Introduction of the Basic Statute of the State, outlining freedoms and restrictions of the press	Abdulaziz bin Mohammed Al-Rawas	Basic Statute of the State (Royal Decree No. 101/1996)
May 29, 1986	Establishment of the Oman News Agency	Abdulaziz bin Mohammed Al-Rawas	Royal Decree No. (39/86)
June 25, 1997	Establishment of Oman Establishment for Press, News, Publishing and Advertising	Abdulaziz bin Mohammed Al-Rawas	Royal Decree No. (43/97)
May 10, 1997	Issuance of the Law on the Control of Artistic Works	Abdulaziz bin Mohammed Al-Rawas	Royal Decree No. (65/97)
November 16, 1997	Abolition of the Oman Authority for Television Art Production	Abdulaziz bin Mohammed Al-Rawas	Royal Decree No. (78/97)
June 10, 2001	Appointment of Hamad bin Mohammed Al-Rashdi as Minister of Information		Royal Decree No. (64/2001)
2004	Lifting of prohibition on private radio and television stations	Hamad bin Mohammed Al-Rashdi	Royal Decree permitting private ownership of broadcast media. Royal Decree No. (95/2004)
2009	Launch of the first private radio station, Hala FM		
September 10, 2011	Amendment of some provisions of the Press and Publications Law	Hamad bin Mohammed Al-Rashdi	Royal Decree No. (95/2011)
October 22, 2011	Amendment of some provisions of Royal Decree No. (108/2010) establishing the Public Authority for Radio and Television and issuing its provisions	Hamad bin Mohammed Al-Rashdi	Royal Decree No. (100/2011)
February 29, 2012	Appointment of Dr. Abdulmunim bin Mansour Al-Hasani as Minister of Information		Royal Decree No. (11/2012)
2016	Suspension of the newspaper Azamn and arrest of journalists following reports on judicial corruption	Dr. Abdulmunim bin Mansour Al-Hasani	Actions taken under the Press and Publications Law.
August 18, 2020	Appointment of Dr. Abdullah bin Nasser Al-Harrasi as Minister of Information		Royal Decree No. (111/2020)
November 10, 2024	Issuance of the new Media Law	Dr. Abdullah bin Nasser Al-Harrasi	New Media Law (Royal Decree No. 58/2024), regulating media activities, rights of media professionals, and penalties for violations.

^*^Compiled by author from Ministry of Information website.

This table lists key legislative milestones and shows how the state aligns media practices with national priorities.

**Table 2:** Major broadcast and media channels in Oman.^*^

**Channel/entity**	**Type**	**Launch date**	**Language**	**Ownership**	**Key notes**
General Radio	Radio	1970	Arabic	State-owned	Launched in Muscat and Salalah; linked by satellite in 1979
General TV Channel	TV	1974	Arabic	State-owned	First national television broadcaster; linked to Salalah by 1979
Oman English FM	Radio	1975	English	State-owned	Among the earliest English-language stations
Shabab Radio Oman	Radio	2003	Arabic	State-owned	Initially broadcast 11 h daily, expanding to 18 h in 2008 and 24-h programming by 2014. It targets youth audiences, reflecting their interests, aspirations, and perspectives
Hala FM	Radio	2007	Arabic	Private	First private radio station in Oman
Hi FM	Radio	2007	English	Private	English-language Top 40; broad national reach
Al-Wisal Radio	Radio	2008	Arabic	Private	Diverse programming; expanded from Muscat to Dhofar
Majan TV Channel	Satellite TV	2009	Arabic	Private	First private satellite broadcaster in Oman
Muscat FM	Radio	2017	Arabic	Private (Muttrah Media)	Known for socially oriented programming, including *timesi.ttfḤawwā' ‘alā al-Hawā'*
T FM	Radio	2018	English	Private	Focused on youth; blends music, dialogue, and current affairs
Shabiba FM	Radio	2018	Arabic	Private	Launched by Space Muscat Media; offers over 28 media products in Arabic and English

^*^Compiled by author from Ministry of Information website.

This table provide an overview of the primary media outlets operating in Oman.

**Table 3:** Major print media outlets in Oman.^*^

**Publication**	**Type**	**Launch date**	**Language**	**Ownership**	**Key features**
*Oman Daily*	Newspaper	1972	Arabic	State-owned	Main official daily; widely circulated among state agencies
*Oman Observer*	Newspaper	1981	English	State-owned	Official English-language daily
*Al-Watan*	Newspaper	1971	Arabic	Private	Oman's oldest daily; maintains pro-government tone
*Oman Tribune*	Newspaper	2006	English	Private (Al-Watan)	Sister paper to *Al-Watan*, aimed at bilingual and expat audiences
*Times of Oman*	Newspaper	1975	English	Private	First English-language daily; known for coverage beyond government narrative
*Al-Shabiba*	Newspaper	1993	Arabic	Private	Sister of *Times of Oman*; Arabic-language youth and local affairs focus
*Al-Zaman* (closed)	Newspaper	2007	Arabic	Private	Notable for critical reporting; permanently shut down in 2016
*Muscat Daily*	Newspaper	2009	English	Private	Known for moderate independence; covered 2011 protests
*The Week* (closed)	Newspaper	2003	English	Private	Free weekly newspaper launched in March 2003

^*^Compiled by author from Ministry of Information website.

This table provide an overview of the primary media outlets operating in Oman.

### 2.2 Theoretical framework

The analysis of women's representation in Omani media draws on three intersecting frameworks, state feminism, neopatriarchy, and symbolic annihilation, each illuminating a different dimension of the relationship between gender, power, and media. Taken together, they reveal how women's visibility is simultaneously produced and constrained by state institutions that deploy empowerment narratives as instruments of legitimacy. State feminism provides a useful entry point for understanding how Gulf governments institutionalize women's empowerment through state-controlled mechanisms that often substitute genuine participation with managed visibility. Originally conceptualized by Hernes (1987, p. 15) as the incorporation of women's interests into official structures, state feminism has been expanded and critiqued by feminist political sociologists such as Moghadam, who argue that when states monopolize women's agendas, activism risks becoming depoliticized and reform symbolic rather than transformative. In the Gulf, this manifests in women's recurrent visibility in ceremonial events, policy imagery, or leadership appointments that signify modernity without challenging underlying hierarchies. As Pitkin (1972, p. 92) distinguishes between symbolic and substantive representation and Fraser (1996, pp. 14–17) warns against “recognition without redistribution,” the Omani and broader GCC cases illustrate how state feminism transforms empowerment into a spectacle of progress rather than a vehicle for equality.

Yet state feminism alone cannot explain how such symbolic empowerment persists. Here, neopatriarchy provides a structural complement. Introduced by Sharabi (1988, p. 7), the concept describes societies that adopt modern bureaucratic institutions while preserving patriarchal authority, creating a system of “distorted modernization.” Although Sharabi's framework has been critiqued for essentialism and for reproducing a West vs. East binary, its structural insights remain analytically useful when stripped of cultural determinism. This study therefore employs neopatriarchy not as a cultural label but as a description of governance structures in which paternalism is embedded within modern institutions. Sharabi's observation that “the state mirrors the patriarchal family” is particularly relevant to Omani media, where protectionist rationales and hierarchical decision-making reproduce gendered dependence. Under such arrangements, modernization coexists with constraint: women are encouraged to participate, but only within boundaries that reaffirm state guardianship and moral order.

However, even this dual framework of institutional co-optation and patriarchal governance must be complemented by an account of representation itself, how women are made visible or erased within discourse. Symbolic annihilation, a concept articulated by Tuchman (1978, pp. 3–8), captures the systematic omission, trivialization, or condemnation of women in media narratives. Foundational feminist media scholarship, particularly Byerly and Ross's critical introduction to women and media (2006), demonstrates how news institutions systematically frame women through stereotypical, apolitical, or domesticated narratives, reinforcing gendered power structures across different media systems (Byerly and Ross, 2006). Subsequent feminist media scholarship has elaborated how newsroom hierarchies and editorial cultures perpetuate this erasure (Byerly, 2011; Ross, 2017, p. 3). In the Arab region, Sakr (2004, 2007) shows that while modernization expands women's visibility, it simultaneously constrains critical debate about gendered power, producing representation that is hyper-visible yet politically mute. Bringing these perspectives together allows a deeper reading of Gulf and Omani media. State feminism explains why visibility is instrumentalized to project state progress; neopatriarchy illuminates how bureaucratic and familial hierarchies sustain that instrumentalization; and symbolic annihilation reveals what these dynamics yield in practice, the selective erasure or trivialization of women's lived realities. Rather than discrete theories, these frameworks intersect as a unified analytic model that problematizes the paradox of Gulf modernity: the coexistence of heightened female visibility and persistent structural inequality. This triadic lens anchors the analysis that follows, tracing how elite narratives reproduce, negotiate, and occasionally resist these interlocking mechanisms of representation and control.

### 2.3 Empirical gaps and the study's contribution

Existing scholarship on Omani media remains sparse and fragmented across political science, communication, and cultural studies. Pre-2020 research primarily addressed women's political participation, religious conservatism, and tribal structures that limit gender inclusion. Recent post-2020 scholarship has expanded to digital media, audience research, and production-side analyses, documenting how self-censorship, institutional hierarchies, and technological mediation shape women's visibility. Yet, few studies integrate elite perspectives on how media governance itself reproduces gendered hierarchies. This article attempts to provide a systematic, elite-based analysis of gender representation in Omani media. By situating elite narratives within the triadic theoretical framework outlined above, it demonstrates how state, culture, and media intersect to sustain symbolic empowerment while restricting substantive gender reform.

## 3 Methodology

### 3.1 Research design, analytic orientation, and participants

This qualitative study is based on 38 semi-structured elite interviews conducted between 2021 and 2025 with Omani journalists, state officials, parliamentarians, academics, activists, and senior media practitioners. These groups were selected because they influence or interpret media governance and public discourse. Following standard definitions in elite interviewing, “elites” are understood as individuals who possess decision-making authority, symbolic influence, or privileged access to institutional power (Harvey, 2011, p. 434). Non-elite voices were excluded to preserve analytical focus on how those shaping narratives perceive gender representation in the media. This choice highlights the discursive production of legitimacy from above, while acknowledging that it does not capture the lived experiences or audience reception of media representations. The study employed an abductive, reflexive thematic design, combining inductive coding with theory-informed interpretation. Abductive reasoning entails an iterative movement between data and theory, where surprising empirical findings prompt theoretical refinement (Timmermans and Tavory, 2012, pp. 170–175). Reflexive thematic analysis (Braun and Clarke, 2006, 2019) involved continuous researcher self-awareness about interpretive influence, theoretical sensitivity, and socio-cultural positioning. The analysis treated state feminism, neopatriarchy, and symbolic annihilation as sensitizing concepts, orienting but not pre-determining interpretation.

### 3.2 Data collection and triangulation

Interviews followed a semi-structured format to balance comparability with flexibility appropriate to elite participants (Brinkmann and Kvale, 2015, pp. 150–156). Guides covered four domains: (1) media regulation and governance, (2) newsroom practices and institutional constraints, (3) gender representation and women's visibility, and (4) reform prospects and policy narratives. Interviews lasted between 45 and 120 min and were primarily conducted in Arabic, some bilingually. Several interviews were audio-recorded with consent, transcribed, and translated using a dual-track procedure, an initial translation reviewed by a second bilingual researcher and back-checked on theoretically salient excerpts to preserve conceptual equivalence (van Nes et al., 2010). Data were anonymized, encrypted, and managed in ATLAS.ti using coded identifiers (e.g., Acad-F-07, “Academic, Female, Participant 7”). To contextualize elite perspectives, five women-focused television programs that were heavily referenced in these interviews were observed and documented through field notes: *Shu'*ū*n ‘*$\bar{\text{A}}$*iliyya, Shaq*ā'*iq, Hay*ā*t'kum, Kay*ā*n, and timesi.ttfḤaww*ā'* ‘al*ā *al-Haw*ā'. These were not subjected to systematic content analysis but served as contextual evidence within data triangulation, enriching understanding of the representational environment described by participants. Supplementary materials such as media laws, national strategies, and regulatory circulars were also reviewed to anchor interview data within Oman's institutional context (Bowen, 2009).

### 3.3 Data analysis

Analysis followed Braun and Clarke's six-phase model: familiarization, coding, theme construction, review, definition, and reporting. Two analysts independently open-coded five transcripts to calibrate a shared codebook before iterative theme development across the corpus. Reflexive thematic analysis emphasizes researcher subjectivity as a resource, not a bias, acknowledging that meaning is co-constructed through interpretation. After inductive theme stabilization, a theory-led reading re-engaged the data through three interpretive lenses: State feminism, examining how women's empowerment is framed as a legitimizing state project. Neopatriarchy, tracing how modernization coexists with patriarchal control. Symbolic annihilation, identifying omission, trivialization, and condemnation in portrayals of women.

### 3.4 Rigor, ethics, and reflexivity

Analytical rigor was ensured through triangulation (interviews, media observation, and document review) and peer debriefing (a volunteer analyst independently coded one-third of the transcripts). An audit trail documented coding decisions and theme evolution. Ethical safeguards followed institutional approval protocols. Informed consent was obtained, and participants were granted graded anonymity, pseudonyms by default, named attribution only upon explicit written consent. Sensitive remarks made off-record were excluded from citation. Reflexivity and positionality were integral to the process. As an Omani woman and gender scholar affiliated with an international university, my position facilitated trust and access but also shaped participants' responses and expectations. To mitigate interpretive bias, I maintained positionality memos reflecting on power asymmetries, insider–outsider dynamics, and cultural sensitivities throughout the fieldwork and analysis stages. Finally, the scope of the study was purposefully delimited to elite perceptions rather than audience reception or systematic content analysis. This lens illuminates how those at the intersection of policy, media, and governance conceptualize women's representation, offering critical insights into the symbolic, institutional, and political dynamics that shape gendered communication in Oman.

## 4 Findings

Drawing on 38 elite interviews and targeted observations of state-owned programs, newspapers coverage of Omani Women's Day and private broadcasts, the analysis identifies three interrelated patterns in how Omani media represent women. First, women's visibility clusters around ceremonial or high-visibility occasions. Second, institutional and technological modernization coexists with regulatory and cultural constraints that temper critical inquiry and rights-based discourse. Third, mainstream media programs routinely omit, trivialize, or stereotype women's everyday experiences. These patterns recur across the corpus, yet respondents' explanations, and the recommendations they propose, diverge according to their institutional roles. State officials frame constraints as stewardship and sensitivity to audience cultural norms; journalists emphasize uncertainty and the risk of sanction; senior broadcasters focus on how gatekeeping operates as daily editorial practice; and academics and civil-society figures interpret these same dynamics as structural features of a managed media and information eco-system. The discussion that follows develops these themes while keeping such divergences in view and concludes with a synthesis that integrates the strands into a coherent account of gendered media governance in Oman.

### 4.1 Women as symbols of state progress (state feminism/symbolic empowerment)

Across the 38 elite interviews, a prevailing narrative emerged that women's visibility in Omani media operates less as evidence of structural change than as a symbolic performance of national progress. Yet this agreement was not absolute. Journalists, state officials, and academics often converged on the idea that gender representation serves the state's image, while diverging on the degree of intent, legitimacy, and consequence whether intended or unintended. These variations reveal how elites interpret women's media visibility through the prism of state feminism: as both a sign of achievement and a mechanism of containment. Participants from governmental and quasi-governmental institutions tended to frame media representations as patriotic, emphasizing unity and national cohesion. A senior official at the Ministry of Social Development (MOSD) described Omani Women's Day as “highly visible yet largely ceremonial,” noting that televised festivities featuring “flowers, gifts, and speeches” function more as “social rituals than policy dialogues.”^1^ For such participants, the visibility of women symbolized stability, a marker of modernization balanced against social conservatism. By contrast, journalists and independent media practitioners viewed these portrayals more critically. A media practitioner at Majlis A'Shura observed that women's representation has narrowed rather than expanded: “10 years ago, there were dedicated programs for women. Today, we no longer find this. Women now appear only in general programs tied to national or international celebrations.”^2^

Similarly, a retired television presenter lamented the reduction of women's programming to lifestyle segments: “In the last 5 years, from 2020 to 2025, there was only one TV program that claimed to target the modern Omani woman, Kayān. But who is this ‘modern woman'? How much of the population does she represent when the program focuses on makeup, home décor, and cooking? It reduces women's contemporary values and potential.”^3^ Similarly, a retired employee at MOSD and a social activist criticized parliamentary awareness programs such as *timesi.ttfṢawtak Sh*ū*r*ā for featuring women merely to avoid criticism rather than as agents of debate: “Women are mentioned only in passing, as part of awareness efforts. They are not treated as political actors in their own right.”^4^ The contrast between bureaucratic pride and journalistic frustration highlights the ambivalence surrounding symbolic empowerment. For officials, celebratory coverage reinforces legitimacy; for practitioners, it exposes the superficiality of inclusion.

Academic respondents, including sociologists and strategic communication scholars, echoed this critique, situating it within broader developmental paradigms. One academic and media commentator summarized: “Representation is still counted as numbers, not normalized. Equality is real only when women's presence is no longer exceptional.”^5^ This statement encapsulates the quantitative logic underpinning state feminism: progress is measured through visible appointments, female ministers, ambassadors, or Oman Council members, rather than through shifts in institutional power or everyday practice. For these elites, the problem is not absence but instrumentalization. Women's achievements are framed as triumphs of the Sultanate, not of women's collective agency, mirroring what Pitkin (1972, p. 60) calls symbolic representation, the appearance of inclusion without commensurate authority.

Notably, several participants within state institutions contested this interpretation, asserting that media's role is not to critique policy but to “highlight the government's efforts.”^6^ Their defense reveals the protective ethos surrounding state-sanctioned media: a belief that public critique risks “being exploited by external actors to damage Oman and deter investors.”^6^ Thus, what academics label symbolic is, for officials, prudent. This divergence underscores how elites' institutional positions shape their perceptions of empowerment, revealing internal pluralism within the elite class rather than uniform endorsement of the state line. When asked to compare Omani portrayals with those in neighboring Gulf states, participants offered mixed assessments. Government actors highlighted the “measured and respectful” tone of Omani media relative to other GCC states more assertive promotional style. Journalists and activists, however, perceived this restraint as another form of control. A social activist observed that “people don't trust government media anymore… private outlets are more trustworthy… and we should have more of these.”^7^

These perceptions resonate with regional scholarship documenting how Gulf regimes deploy women's visibility to affirm modernization while deflecting attention from structural inequality (Al-Rasheed, 2013, pp. 122–125). Omani elites' commentary thus situates the national media within a familiar Gulf grammar of reform: women appear as ambassadors of progress, yet the boundaries of that visibility remain tightly drawn. Despite widespread critique, a minority of participants, particularly senior bureaucrats and older media figures, interpreted ceremonial visibility as a legitimate stage in an evolutionary process. One senior editor defended the current trajectory: “Every society needs time. The important thing is that women are present in the image of Oman. That in itself breaks stereotypes.”^8^ This pragmatic view coexists uneasily with younger journalists' impatience for substantive transformation. The generational divide underscores differing expectations: older elites prioritize symbolic continuity and gradual reform; younger ones advocate for open debate and accountability. Yet both acknowledge the constraints of a system in which visibility is politically mediated.

Across these narratives, a recurring pattern emerges: women's visibility functions as an index of good governance and national advancement. Whether in televised tributes, development campaigns, or parliamentary coverage, empowerment is celebrated in abstract terms, detached from the everyday realities of legal inequity, workplace discrimination and harassment, or access to leadership. Several respondents explicitly linked this to the post-2011 consolidation of media control, noting that gender became part of a broader “harmonization” of public discourse. A senior policy expert noted that coverage is ‘timid and not rights-based… framed as services granted out of generosity and honor': “After 2011, all sensitive themes, tribalism, religion, gender, were reframed under ‘development.' It is easier to speak of progress than of problems.”^9^ Such statements reveal how state feminism operates discursively through the language of modernization: gender equality is invoked to signify administrative efficiency and international alignment, not to challenge domestic hierarchies.

These tensions suggest that elite discourse is itself a site of negotiation, one that mirrors the very dynamics of controlled pluralism characterizing Omani public life. Overall, the theme of women as symbols of state progress illustrates the operational logic of state feminism in Oman: empowerment is visualized, quantified, and celebrated, but rarely politicized. The persistence of this paradigm points to the resilience of a developmentalist media culture born during the post-Dhofar nation-building era and sustained by the ethos of harmony over contestation. Women's representation continues to serve as an emblem of stability and legitimacy, an aesthetic of equality that reassures rather than transforms.

### 4.2 Modernization without liberation (neopatriarchy)

The second major theme emerging from the interviews concerns the paradoxical coexistence of modernization discourse and patriarchal control in Oman's media and governance landscape. Participants from across government institutions, journalism, academia, and civil society repeatedly described an environment where progressive rhetoric on women's empowerment coexists with rigid social hierarchies and legal ambiguity. Yet their interpretations of this paradox diverged sharply depending on institutional role and generational outlook.

Government-affiliated elites, particularly policy advisers and former bureaucrats, tended to frame restrictive laws and regulatory systems as necessary safeguards for national cohesion. An ex- parliamentarian acknowledged the constraints but defended them as “protective rather than punitive”: “Omani journalism must reflect our social values. There are limits, but these limits maintain dignity and social peace. Not every issue needs to be discussed in public.”^8^ By contrast, independent media figures and academics regarded these same frameworks as symptomatic of deeper patriarchal governance. A policy expert and consultant emphasized that institutional conservatism was not merely cultural but structural: “Our legal framework still sees women as dependents, not as full citizens. The problem is not religion; it is the bureaucratic mindset that assumes women always need guardianship.”^9^ Several journalists highlighted how the 2013 law protecting *haybat al-dawla* (“the majesty of the state”) serves as an elastic tool for silencing gender-related critique. As one explained,

“Omani journalists rarely investigate; they fear overstepping vague laws. The 2013 law forbids any discussion that might ‘harm the majesty of the state,' yet no one can define what that means. Because of these elastic boundaries, women's issues appear in the media only timidly and never from a rights-based perspective. They are framed as services granted out of generosity or honor, not as legal entitlements.”^10^

Where policy elites saw prudence, journalists saw intimidation. The divergence illustrates how neopatriarchal governance operates through institutionalized caution: legal ambiguity cultivates self-discipline among media professionals, reinforcing deference to authority while maintaining a façade of modernity. Accounts from radio and television professionals further illuminated how state modernization projects, digital expansion, new infrastructures, and gender-inclusive employment, coexist with pervasive censorship. A current radio presenter recalled directives during the 2021 Sohar protests: “The Ministry of Information sent a written memo to private radio stations forbidding any discussion of layoffs. Even Majlis Ashura members could appear only with prior approval.”^11^ Similarly, another journalist described restrictions during the COVID-19 pandemic: “Officials told us not to discuss domestic violence; it would ‘damage the image of a virtuous society.' Women's suffering disappeared from the airwaves overnight.”^12^ These testimonies reveal the fragility of editorial independence under the veneer of modernization. New platforms and technologies expand the reach of state messaging but not its ideological boundaries. Generational differences also emerged as a key axis of divergence. Older elites, both men and women who came of age during the state-building decades, often justified gradualism and restraint as culturally necessary. A former woman State Council member stated: “Women's political participation will expand, but first we must overcome the economic crisis. Social reform comes after stability.”^13^ Journalists, human rights activists and academics contested this logic, arguing that such caution perpetuates inequality. One mid-career professional expressed frustration: “There are red lines from the Ministry of Information and a fluctuation in the ceiling of freedoms, we don't know whether we're allowed to speak freely or not.”^14^ This divide highlights how neopatriarchy reproduces itself through socialization into bureaucratic paternalism. Senior elites internalize the logic of protection and stability, while younger professionals increasingly question its legitimacy.

Across the interviews, elites described a pervasive culture of anticipatory obedience in Omani newsrooms. Editors and producers routinely pre-empt state intervention through self-censorship. An independent journalist narrated one such episode: “When I wrote about the Suhar protests, the Undersecretary called me and asked to delete the article. He said these were the Minister's instructions. I refused, but afterward I was told my media permit could be withdrawn. The audience never knows what happens behind the scenes.”^15^ Such experiences demonstrate how power operates through informal yet effective mechanisms. Censorship rarely requires explicit suppression; rather, it is sustained by professional norms of loyalty, the fear of losing licenses, and the moral discourse of protecting social harmony. Elites differed on where to locate the roots of this control. Some attributed it to enduring cultural conservatism, while others emphasized bureaucratic inertia. A young public policy specilist and a radio presenter argued: “official media treats problems as ‘developmental' and avoids real causes, economic, social, or political… discussions rely on the wrong approaches and neglect the roots.”^16^ Conversely, a senior official countered that social acceptance, not bureaucratic will, remained the limiting factor: “There is movement from the government, but society needs preparation; when announcements come without preparing people, backlash follows, once clarified, things calm down.”^17^ This tension between culturalist and structural explanations recurred throughout the interviews, reflecting the multiple scales at which neopatriarchy is reproduced. Media policy thus becomes the site where cultural values and bureaucratic paternalism converge: the moral discourse of protecting tradition merges with the administrative discourse of preserving order. A recurrent thread in both official and journalistic narratives was the moralization of loyalty. Women's visibility in the media was often described as a “gift” from the state, contingent upon good behavior and patriotic alignment. A social activist explained: “We are encouraged to speak about success and family values but not about injustice. To stay visible, we must stay positive. Official media is extremely superficial; all we hear is that ‘women have many achievements”' (see text footnote 5). Her comment encapsulates the moral contract at the heart of neopatriarchal modernization: the state celebrates women as symbols of progress while circumscribing their agency through discourses of gratitude and respectability. Visibility becomes conditional upon loyalty, and critique is recoded as ingratitude.

Participants also emphasized that technological and institutional modernization, the expansion of digital newsrooms, training programs, and international partnerships, has not translated into ideological openness. A media entrepreneur summarized the contradiction: “We have fancy studios, but third-world freedom. The structure changed, not the mindset. We don't have ‘media'; we have government propaganda funded by the state, which therefore controls it absolutely.”^18^ Several respondents linked this stagnation to the aftermath of the 2011 Arab uprisings, when the Omani state tightened control over civil-society organizations and public debate. While economic diversification and women's employment were prioritized under Vision 2040, editorial independence remained closely monitored. An ex- parliamentarian reflected: “the Press and Publications Law empowered the Minister [of Information] to decide what even private channels can air… during the Suhar protests, media were prevented from covering the public matter.”^19^ Thus, modernization operates as a performative signifier rather than an emancipatory process, a hallmark of what Sharabi (1988, pp. 5–11) termed modernity without liberation. Despite their institutional differences, most elites converged on one fundamental perception: that modernization in Oman is real but delimited. However, their accounts diverged on whether these limits stem from prudence or oppression. Government officials and long-serving administrators viewed constraint as a stabilizing virtue; journalists, scholars, and civil society actors perceived it as systemic stagnation. Some women officials themselves oscillated between pride and frustration, grateful for visibility yet wary of tokenism. This ambivalence exposes the negotiated nature of gender politics within the elite sphere itself, where individuals simultaneously reproduce and contest patriarchal rationales. Taken together, these testimonies illustrate how Oman's modernization has produced a paradoxical order, an outwardly progressive system underpinned by paternalistic governance. State discourse celebrates women's education, entrepreneurship, and professional success, yet the same institutions enforce behavioral discipline and moral oversight. The elasticity of laws protecting “public morals” and “the majesty of the state” creates a climate in which journalists, editors, and broadcasters internalize constraint as professional responsibility. Modernization becomes both a goal and a control mechanism: it legitimizes authority while re-inscribing obedience. While elite participants differed in emphasis, some defending gradualism, others decrying stagnation, all located women's empowerment within a logic of conditional citizenship. Women's advancement remains contingent on respectability, loyalty, and silence on systemic inequities. This is the essence of Oman's neopatriarchal bargain: a negotiated coexistence of modern infrastructure and patriarchal governance, in which technological progress and professional inclusion mask enduring asymmetries of power.

### 4.3 Silencing and stereotyping (symbolic annihilation)

The third thematic strand concerns the processes of silencing, stereotyping, and trivialization that define women's representation in Omani media. These findings correspond to Tuchman's (1978) notion of symbolic annihilation, where women are not necessarily absent but are rendered invisible through omission, distortion, or reductive portrayals. Participants across institutional sectors converged on the observation that women's realities, particularly issues of violence, discrimination, and workplace inequality, are rarely addressed in depth. However, their explanations for this erasure diverged sharply. Government representatives largely downplayed the existence of gender-based issues, framing discussions of violence or discrimination as unnecessary or even detrimental to national unity. Several officials described women's conditions in Oman as “stable” and “protected,” asserting that “violence does not occur in our society” and that addressing such topics publicly could “distort the country's image.” In this framing, silence is recast as patriotism: acknowledging structural or domestic forms of gender inequality is perceived as damaging to the “national image” and contrary to the media's role in promoting cohesion. By contrast, journalists, academics, and civil-society figures interpreted this avoidance as a deliberate mechanism of censorship and self-censorship, one that sustains patriarchal continuity under the rhetoric of modernization.

Officials within the Ministry of Social Development (MOSD) emphasized that the current content strategy aims to “celebrate success stories and avoid social polarization.” An employee at MOSD defended this approach, explaining that “television should motivate, not scandalize,” though she simultaneously lamented the narrowing of subject matter: “Government channels focus on cooking or beauty segments. When women are mentioned in serious contexts, it is only during elections or seasonal campaigns.”^20^

In contrast, journalists and cultural producers critiqued such selectivity as a structural form of erasure. A prominent poet and columnist recalled how earlier programming was more socially engaged: “Programs like *Shaq*ā'*iq* once discussed women's issues with depth. Now, women's health or education challenges are rarely covered. They are invisible.”^21^ These perspectives expose a clear generational and institutional divide: while government-affiliated elites emphasize harmony and national cohesion, independent practitioners lament the loss of spaces where gendered social issues could be addressed with nuance.

Multiple respondents, identified entertainment programming as a site where patriarchal stereotypes persist. An expert on women's affairs at MOSD explained: “Dramas and sitcoms still show women as weak, emotional, or broken. We never see women as scientists or decision-makers.”^22^ Media practitioners corroborated this observation, arguing that female characters are written to embody “virtue” and “sacrifice,” reflecting broader cultural expectations of femininity. Some producers justified this as catering to audience preference, while others admitted that scripts undergo informal censorship before airing. The persistence of such representations underscores how symbolic annihilation operates not through absence alone but through the reproduction of limiting archetypes.

Several participants recounted experiences of direct interference in the framing of women's issues. A former television presenter described how an academic guest on a domestic violence segment “reversed her own study findings under pressure,” publicly denying that violence existed despite privately acknowledging widespread cases, including incest. The presenter reflected: “Media pressure forces women to deny truth. We all know the reality, but on air, truth becomes dangerous” (see text footnote 14). A civil-society representative captured the systemic dimension of this process: “The job of government media is to pacify society. Even women journalists protect themselves by avoiding sensitive topics. No one wants to risk their job or reputation.”^23^ Such testimonies reveal that symbolic annihilation is not simply the product of patriarchal ideology but also of institutionalized self-preservation, what several participants called “survival journalism.” Academics and activists highlighted an emerging paradox: as mainstream media suppresses critical discussion, digital platforms have become alternative arenas for women's expression. A former university dean reflected on the gendered silences surrounding the 2021 Sohar protests and the economic downturn: “Men dominated the demonstrations, though most job-seekers were women. Social norms kept women from protesting, but online there was bold feminist mobilization. Digital platforms became a culturally safe zone for women to express frustration. Still, mainstream media ignored this activism, focusing only on male protesters and official statements.”^24^ Her account suggests that while women's agency persists, it is displaced from public broadcast to the more ambiguous terrain of social media. This digital migration reinforces Tuchman's observation that symbolic annihilation operates through omission: women's participation is not extinguished but rendered invisible within official discourse.

When asked who bears responsibility for these silences, elites offered divergent explanations. Government officials attributed the problem to “insufficient training” among journalists and “public readiness,” implying that audiences themselves resist more assertive gender coverage. Journalists, conversely, pointed to structural censorship and the absence of editorial autonomy. A retired editor and an owner of a private media training center stated: “We used to try to cover issues like harassment or pay inequality, but stories now are killed before publication. The justification is always the same: ‘society isn't ready.' But who decides that? The state or the people?” (see text footnote 3).

Academics interpreted this deflection as part of a broader cultural economy of responsibility, where accountability is diffused among institutions to avoid reform. The circular logic, “the people aren't ready” because they are never exposed to open debate, perpetuates a managed silence that normalizes exclusion. Several senior female journalists and broadcasters described the emotional toll of working within such restrictive environments. One senior media figure recounted that she was repeatedly instructed to soften her tone and avoid appearing “political,” noting that the cumulative pressure to self-moderate was exhausting. Others described subtler forms of silencing through moral scrutiny and class hierarchy. Women from prominent families often enjoyed greater freedom to speak, while those from modest or rural backgrounds faced closer monitoring. These differences reveal that symbolic annihilation intersects not only with gender but also with class and social capital within elite structures.

Despite differences in tone, a general consensus emerged among elites that the current media environment does not adequately represent women's lived realities. Yet disagreement persisted over causality. Government officials attributed this to audience conservatism and cultural sensitivity; journalists and academics framed it as systemic censorship; and activists viewed it as deliberate marginalization. These varying interpretations underscore the fragmented nature of elite discourse: while all recognized that women's voices are constrained, they disagreed on whether reform should come from within institutions or from broader social transformation.

A senior producer described this as “calendar feminism,” explaining that empowerment becomes “a seasonal performance rather than a continuous conversation.”^25^ Academic participants similarly linked this to institutional inertia: ministries and broadcasters operate on short-term initiatives rather than coherent strategies. The absence of long-term planning translates into fragmented programming, where achievements are highlighted but structural inequalities, such as workplace harassment, inheritance disputes, or access to leadership, remain unaddressed. Participants further identified the centralized control of the Ministry of Information as a core barrier to reform. Journalists noted that even private outlets depend on government advertising and licensing, reinforcing economic dependency and discouraging dissent. As one independent editor put it, the financial and regulatory reliance on state structures constrains critical coverage. This structural dependency mirrors the broader political economy of Gulf media systems, where modernization proceeds under the shadow of state oversight. Female media professionals from older generations provided some of the most poignant testimonies about the personal cost of these hierarchies. Several recounted experiences of professional rivalry and isolation within gendered institutions. One prominent broadcaster shared: “My director came from a very prominent family, and my program was popular with the public. After 2011, the Minister asked me to host a discussion program, but my director filed a false case against me. I had to defend myself before the Minister. He understood, but the experience left scars.”^3^ Her account illustrates how patriarchal power is not only exercised through male dominance but also reproduced within elite female competition, where class and connections shape opportunities for visibility. These experiences reveal how structural neopatriarchy is reinforced through interpersonal dynamics within the media field itself.

## 5 Discussion and implications

This study demonstrates that women's representation in Omani media reflects a deliberate coexistence of visibility and constraint. Rather than signaling straightforward progress or regression, such portrayals constitute a managed equilibrium, a strategic balance through which inclusion reinforces legitimacy. Elite perspectives reveal that gender representation functions not merely as discourse but as a technology of governance. Through the triangulation of state feminism, neopatriarchy, and symbolic annihilation, this analysis shows how modernization, patriarchy, and moral authority intersect within Oman's media system to sustain controlled pluralism and preserve state authority.

The Omani case extends debates on state feminism by illustrating how empowerment rhetoric becomes institutionalized as a mode of governance. As Hernes (1987) and Moghadam (2005) argue, state feminism bureaucratizes gender equality, transforming social demands into technocratic management. In Oman, women's appointments and visibility in official media project reform while avoiding redistributive change. Elite accounts clarify this transformation: government officials equated women's visibility with national unity, while journalists and academics perceived it as performative, a legitimizing spectacle rather than substantive empowerment. This tension echoes Fraser's (1996) critique of “recognition without redistribution,” where symbolic inclusion substitutes for material justice. Yet several female officials viewed state-backed empowerment as a pragmatic route to incremental gains, suggesting that co-optation and progress coexist. These dynamics define what can be termed authoritarian developmental feminism, a governance model merging inclusionary rhetoric with paternalistic oversight. In this framework, women are made visible as symbols of national advancement but constrained as autonomous political actors. Legitimacy, therefore, is constructed through the careful management of gendered participation.

Sharabi's (1988) notion of neopatriarchy, modernization without emancipation, gains renewed relevance when examined structurally rather than culturally. In Oman, patriarchal authority endures not simply through tradition but through institutional design: legal ambiguities, moral codes, and bureaucratic hierarchies that sustain dependence and discipline. Laws safeguarding *haybat al-dawla* (“the majesty of the state”) and editorial norms of “respectability” function as instruments of administrative patriarchy, cultivating what Joseph (1996) describes as relational governance, wherein the state mirrors the patriarchal family's protective hierarchy. Elite testimonies illustrate how this control is internalized: journalists framed censorship as professionalism, while senior women often reproduced paternalistic expectations toward younger colleagues. These dynamics align with Kandiyoti's (1988) concept of the patriarchal bargain, a negotiated stability in which women's advancement is contingent upon loyalty and conformity to patriarchal authority. In Oman, women's symbolic visibility and educational success coexist with legal and institutional restrictions that delimit their public agency. Neopatriarchy thus operates not only as male dominance but as a socially internalized system of obedience, sustained by negotiated consent and moral discourse. Modernization, digital expansion, and professionalization reinforce rather than disrupt these hierarchies, institutionalizing loyalty and embedding patriarchal authority within bureaucratic rationality. The result is a bureaucratic patriarchy in which gender reform and censorship coexist as mutually sustaining mechanisms of moral governance, what might be termed modernization without liberation.

Tuchman's (1978) concept of symbolic annihilation, the erasure or trivialization of women in media, captures how omission becomes a moralized form of control. Elite narratives revealed that avoiding sensitive topics such as domestic violence or workplace discrimination was often justified as protecting the “national image.” This selective silence transforms censorship into virtue, depoliticizing women's suffering in the name of cohesion. Such erasure is neither passive nor accidental but enacted through editorial directives and self-censorship. Visibility is further stratified by class and geography: elite women appear as icons of national progress, while working-class and rural women remain invisible. Even digital platforms reproduce this dynamic, amplifying celebratory imagery while filtering critique, rendering visibility itself a regulated performance. Symbolic annihilation, therefore, extends beyond representation to operate as an apparatus of legitimacy, through which moral order is performed and dissent contained.

Elite discourse is far from homogeneous. Divergences emerged along generational, institutional, and gendered lines: government actors emphasized gradual reform and cultural preservation; journalists and academics critiqued bureaucratic censorship; and younger civil-society figures linked silence to systemic inequality. These distinctions show that elites are co-producers of legitimacy, negotiating between loyalty, reform, and belief rather than merely reproducing state ideology. Kandiyoti's (1988) concept of the patriarchal bargain helps interpret these negotiations. For many senior women in media, empowerment operates as an institutionalized bargain, visibility and recognition exchanged for loyalty and discretion. This feminized professionalism, marked by moral restraint and emotional labor, sustains both institutional reputation and patriarchal order. Yet this bargain is evolving. Younger professionals increasingly frame empowerment as authorship and participation, not symbolic inclusion, suggesting subtle generational shifts within the elite sphere. Despite enduring hierarchies, the findings reveal spaces of negotiation and potential transformation. Younger elites' emphasis on digital participation, cross-sector collaboration, and culturally grounded reform reflects what Abu-Lughod (1998) describes as *everyday forms of negotiation*, subtle acts through which women work within existing structures to create change without overt confrontation. Change may thus unfold through gradual pluralization rather than rupture. Structurally, meaningful reform requires institutional independence, ethical media regulation, and diversified leadership that includes women from varied social and regional backgrounds. Symbolic inclusion must evolve into substantive participation, where women not only appear in media but shape its agendas. Integrating state feminism, neopatriarchy, and symbolic annihilation provides a cohesive framework for understanding this tension between control and reform. It clarifies how Gulf modernization operates through the calibration of visibility and legitimacy, producing a media order that is both progressive and restrictive, celebratory and surveilled. Within this framework, elite perceptions serve as the key to understanding how gender, authority, and moral governance are continually negotiated in contemporary Oman.

## 6 Conclusion

This study demonstrates that Omani elites overwhelmingly perceive women's representation in the media as symbolic, patriarchally constrained, and stereotypical. While women frequently occupy the spotlight in celebratory contexts such as Omani Women's Day, national achievements, or international recognition campaigns, their everyday struggles with legal inequities, workplace discrimination, and underrepresentation in leadership remain marginal. Visibility, therefore, functions less as empowerment than as an instrument of image management. Media institutions reproduce a narrative in which women's progress signifies national advancement, but within boundaries that preserve moral order and political stability. The result is a system where empowerment is celebrated rhetorically yet constrained institutionally. The findings reveal that women's visibility in Omani media primarily serves projects of state legitimacy. Governance structures, shaped by censorship, licensing oversight, and hierarchical bureaucracies, restrict editorial autonomy and reinforce paternalistic gatekeeping. Simultaneously, the silencing and trivialization of women's voices through entertainment stereotypes or selective omission expose the deeper contradictions of the empowerment discourse. Structural inequalities are acknowledged but rarely interrogated; women's achievements are showcased, yet their rights-based struggles remain largely invisible.

The Omani case mirrors broader Gulf and Arab regional dynamics in which media operates as a vehicle of state-led modernization, legitimizing authority through controlled narratives of gender inclusion while avoiding substantive redistributive reform. Neighboring states such as Saudi Arabia and the UAE have adopted more assertive media strategies to project images of rapid transformation and female leadership, yet Oman's approach remains notably cautious, favoring gradualism, cultural preservation, and consensus over critical engagement. As elite participants repeatedly emphasized, this caution leaves Omani media detached from lived realities and ill-equipped to function as a platform for dialogue on gender equity or civic accountability.

The policy and reform recommendations proposed in this study derive from both empirical findings and the suggestions voiced by elite participants. Interviewees, including journalists, parliamentarians, and policymakers, identified editorial independence, professional safeguards, and inclusivity in decision-making as essential steps toward meaningful reform. These insights are further supported by the study's broader analytical findings, which show how structural control and institutional gatekeeping limit women's representation. For Omani media to move beyond symbolic representation toward substantive empowerment, reform must therefore address both structure and ethos. Editorial independence requires institutional guarantees of press freedom, professional protection mechanisms for journalists, and diversification of ownership models to reduce financial dependence on state institutions. Equally vital is the systematic inclusion of women in decision-making roles across editorial boards, regulatory councils, and production management. Representation must also extend beyond urban elites to include marginalized and rural women, whose realities remain largely absent from national narratives. Sustained, year-round programming, rather than event-based coverage, should ensure depth and continuity in addressing women's legal, economic, and social issues.

Several interviewees pointed to digital media as an emerging space of cautious innovation, suggesting that online platforms can expand pluralism if accompanied by ethical moderation, equitable access, and content diversification. Building on these perspectives, the study finds that digital engagement could enable more interactive dialogue between citizens and policymakers, transforming media from a broadcast of authority into a participatory public sphere. In this respect, elite interviews reveal cautious optimism: while participants acknowledged systemic constraints, they also expressed an emergent will for reform and a desire to harmonize Oman's cultural and religious values with universal principles of justice, dignity, and equality.

Women's representation in Omani media thus remains more a reflection of state priorities than a mirror of social realities, yet it also contains the seeds of transformation. By cultivating long-term, coherent strategies that align empowerment with developmental and ethical imperatives, Omani media could transcend symbolic performance and assume a transformative role in shaping an inclusive national future.

This research makes three interrelated contributions. First, it provides the first systematic, elite-based analysis of women's media representation in Oman, filling a gap in Gulf gender and media scholarship that has traditionally focused on audience reception or textual analysis. By centering the voices of those who produce, regulate, and interpret media, parliamentarians, journalists, academics, and civil-society figures, it reveals how structural, cultural, and generational forces interact to shape gender discourse and sustain legitimacy. Second, it advances theoretical debates by integrating state-driven empowerment, patriarchal governance, and symbolic marginalization into a unified explanatory model. This triadic framework, linking state feminism, neopatriarchy, and symbolic annihilation, captures the coexistence of progress and control that defines authoritarian modernization projects. Third, it offers empirically grounded, policy-oriented insights for reform: the need for editorial autonomy, sustained gender-sensitive programming, professional development, and inclusive governance structures that embed women's perspectives into institutional decision-making.

Future research could build on these findings by comparing elite perspectives across Gulf states to trace regional convergences and divergences in gender representation under evolving reform agendas. Cross-national studies could examine how media strategies intersect with national visions such as Oman's Vision 2040, Saudi Arabia's Vision 2030, and the UAE's Vision 2021 in constructing narratives of women's empowerment. Further exploration of digital media and grassroots activism would illuminate how alternative communication spaces negotiate, resist, or reproduce patriarchal norms. Longitudinal studies of elite generational change could also reveal whether emerging cohorts of journalists and policymakers will sustain, reform, or transcend the patriarchal bargains of their predecessors. In sum, this study situates Omani media at the crossroads of modernization and moral governance, where women's empowerment is both mobilized and managed. By integrating elite perspectives with structural and theoretical analysis, it shows that understanding how elites construct and rationalize legitimacy is essential to envisioning a media landscape that reflects not only the nation's aspirations for progress but also its capacity for justice, equality, and genuine public dialogue.

## Data Availability

The original contributions presented in the study are included in the article/Supplementary material, further inquiries can be directed to the corresponding author.

## Footnotes

1 MOSD-F-34, Muscat, 2022.

2 Jour-F-03, Muscat, 2022.

3 Jour-F-38, Muscat, 2025.

4 MOSD-F-08, Muscat, 2023.

5 Acad-F-30, Muscat, 2023.

6 Parl-M-16, Muscat, 2023.

7 CS-F-20, Muscat, 2023.

8 Jour-F-31, Muscat, 2023.

9 Gov-M-01, Muscat, 2023.

10 Jour-M-02, Suhar, 2021.

11 Jour-M-37, Suhar, 2024.

12 Jour-F-09 and Jour-F-10, Muscat, 2022.

13 Parl-F-06, Muscat, 2023.

14 Acad-F-29, Muscat, 2023.

15 Jour-M-02, Muscat, 2021.

16 Jour-M-23, Muscat, 2023.

17 Parl-F-22, Muscat, 2023.

18 Jour-M-32, Muscat, 2023.

19 Parl-M-15, Muscat, 2023.

20 MOSD-F-28, Muscat, 2023.

21 Jour-F-25, Muscat, 2023.

22 MOSD-F-19, Muscat, 2021.

23 CS-F-12, Muscat, 2023.

24 Acad-F-35, Muscat, 2022.

25 Jour-F-31, Muscat, 2022.
