# Supplementary material for: Elites' perceptions of women's representation in the Omani media
Source: Front Sociol. 2025 Nov 27;10:1724294. doi: 10.3389/fsoc.2025.1724294 (PMC12697205; doi:10.3389/fsoc.2025.1724294)
Supplement: Supplementary file 1 [file Table_1.docx]

**Appendix A:**

This table presents anonymized information about the 38 elite interviewees who participated in the study between 2021 and 2025. Codes follow the format [Role]-[Gender]-[Number] to indicate each participant’s sector, gender, and sequence of interview, without revealing any personally identifiable information. The table summarizes professional background, interview year, and location to preserve analytical transparency while ensuring confidentiality.

Table A1. Key Table of Interview Participants (Anonymized)

| **Code** | **Role / Sector** | **Gender** | **Interview Year** | **Location** |
| --- | --- | --- | --- | --- |
| **Gov-M-01** | A Policy expert and social activist | Male | 2023 | Muscat |
| **Jour-M-02** | An independent journalist / Media practitioner | Male | 2021 | Suhar |
| **Jour-F-03** | Media practitioner  (Majlis A’Shura) | Female | 2022 | Muscat |
| **Parl-M-04** | Parliamentarian (Majlis A’Shura) | Male | 2023 | Muscat |
| **Parl-M-05** | Parliamentarian (Majlis A’Shura) | Male | 2023 | Muscat |
| **Parl-F-06** | Parliamentarian (State Council) | Female | 2023 | Muscat |
| **Gov-F-07** | Government advisor | Female | 2022 | Muscat |
| **MOSD-F-08** | Media officer / Social Development | Female | 2023 | Muscat |
| **Jour-F-09** | Writer and media practioner | Female | 2022 | Muscat |
| **Jour-F-10** | Entrepreneur and media practitioner | Female | 2022 | Muscat |
| **Jour-F-11** | Media practioner / Policy consultant | Female | 2022 | Muscat |
| **CS-F-12** | Civil society representative | Female | 2023 | Muscat |
| **Parl-F-13** | Parliamentarian | Female | 2023 | Muscat |
| **Parl-M-14** | Parliamentarian | Male | 2023 | Muscat |
| **Parl-M-15** | Parliamentarian | Male | 2023 | Muscat |
| **Parl-M-16** | Parliamentarian | Male | 2023 | Muscat |
| **Activ-M-17** | Civil society activist and social media Influencer | Male | 2023 | Muscat |
| **Gov-F-18** | Government professional | Female | 2023 | Muscat |
| **MOSD-F-19** | Expert / Ministry of Social Development | Female | 2021 | Muscat |
| **CS-F-20** | NGO leader | Female | 2023 | Muscat |
| **CS-F-21** | NGO activist | Female | 2023 | Muscat |
| **Parl-F-22** | Parliamentarian / NGO founder | Female | 2023 | Muscat |
| **Jour-M-23** | Economist and media prctioner / Policy researcher | Male | 2023 | Muscat |
| **Parl-F-24** | Parliamentarian | Female | 2023 | Muscat |
| **Jour-F-25** | Media practioner and a writter | Female | 2023 | Muscat |
| **Gov-M-26** | A judge | Male | 2023 | Muscat |
| **CS-F-27** | Civil society advocate | Female | 2023 | Muscat |
| **MOSD-F-28** | Media officer / Ministry official | Female | 2023 | Muscat |
| **Acad-F-29** | Human rights expert | Female | 2023 | Muscat |
| **Acad-F-30** | Academic / Media commentator | Female | 2023 | Muscat |
| **Jour-F-31** | Media prationer and a ex parliamentarian | Female | 2023 | Muscat |
| **Jour-M-32** | Media prationer and a ex parliamentarian | Male | 2023 | Muscat |
| **Jour-F-33** | A retired media prationer | Female | 2023 | Muscat |
| **MOSD-F-34** | Senior official/ Ministry of Social Development | Female | 2023 | Muscat |
| **Acad-F-35** | Academic | Female | 2023 | Muscat |
| **Acad-M-36** | Academic / Political historian | Male | 2023 | Muscat |
| **Jour-M-37** | Media Practioner | Male | 2025 | Muscat |
| **Jour-F-38** | A retired media prationer/ Consultant | Female | 2025 | Muscat |

**Ethical note:**

All participants provided informed consent under the ethical approval granted by the author’s institution. To safeguard confidentiality, particularly given the political sensitivity of media and gender discussions in Oman, all names have been replaced with coded identifiers. Roles, sectors, and interview contexts are described in generalized form to prevent deductive disclosure. Where participants explicitly authorized attribution, statements remain paraphrased to maintain anonymity. This approach follows the Frontiers in Sociology policy on participant protection and the Committee on Publication Ethics (COPE) guidelines for qualitative research involving identifiable elites.
